# Refractory ulcers of both legs with psoriasis vulgaris successfully treated with dehydrated human amnion/chorion membrane: A case report

**DOI:** 10.1016/j.jpra.2025.02.015

**Published:** 2025-02-26

**Authors:** Soma Nakaso, Hyakuzoh Ueda, Chiemi Kaku, Yuki Ideguchi, Aya Miyama, Rei Ogawa

**Affiliations:** a Department of Plastic and Aesthetic Surgery, Shonan Kamakura General Hospital, Kanagawa, Japan; b Department of Plastic, Reconstructive and Aesthetic Surgery, Nippon Medical School, Tokyo, Japan

**Keywords:** Psoriasis vulgaris, Refractory ulcers, Human amnion/chorion membrane

## Abstract

Patients with psoriasis are particularly susceptible to skin damage and secondary infections due to impaired skin defenses and chronic inflammation. This case report describes an 80-year-old male with psoriasis vulgaris and a refractory venous stasis ulcer that successfully achieved epithelialization following treatment with dehydrated human amnion/chorion membrane (dHACM; EPIFIX®). Despite initial management involving infection control and wound debridement, the ulcer exhibited minimal improvement until the application of dHACM. Within 4 wk, substantial wound contraction and epithelialization were achieved, eliminating the need for skin grafting. The regenerative properties of dHACM, employed in accordance with the TIMERS framework, facilitated effective wound healing through a minimally invasive approach. This case represents the first documented use of dHACM for a venous ulcer in a patient with psoriasis vulgaris, underscoring its potential as an innovative therapeutic option for managing chronic ulcers.

## Introduction

Patients with psoriasis are predisposed to skin damage and secondary infections owing to compromised skin defences and chronic inflammation. Consequently, a risk of cellulitis progression exists. Here, we present a case of an elderly male with psoriasis vulgaris who achieved successful epithelialization of a refractory venous stasis ulcer using dehydrated human amnion/chorion membrane (dHACM; EPIFIX®, MiMedx Group Inc. Marietta, GA, USA). The patient underwent infection control, wound bed preparation, and tissue regeneration using dHACM in accordance with the TIMERS framework,^1^ which resulted in remarkable wound healing with conservative treatment. This case report further discusses previous studies that examined the efficacy of dHACM.

## Case report

**Figure 1:**
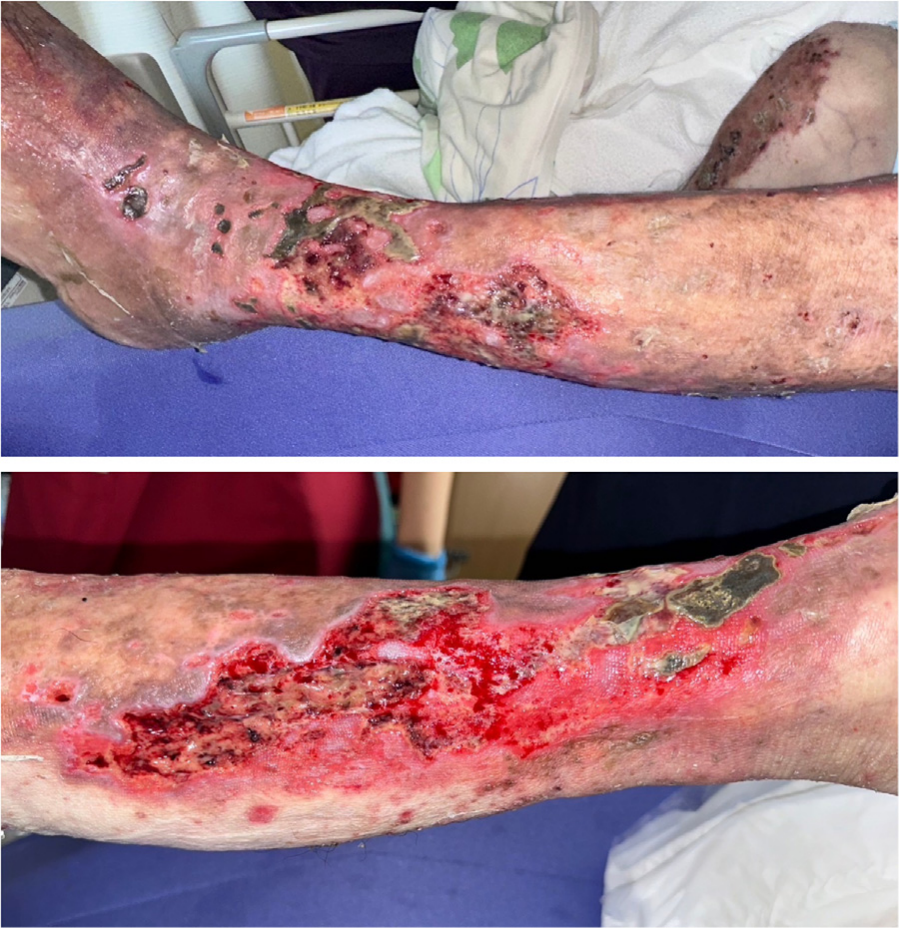
Initial presentation. Ulcers resulting from congestive dermatitis cover over half of the lower leg, with necrotic tissue overlaying these ulcers.

**Figure 2:**
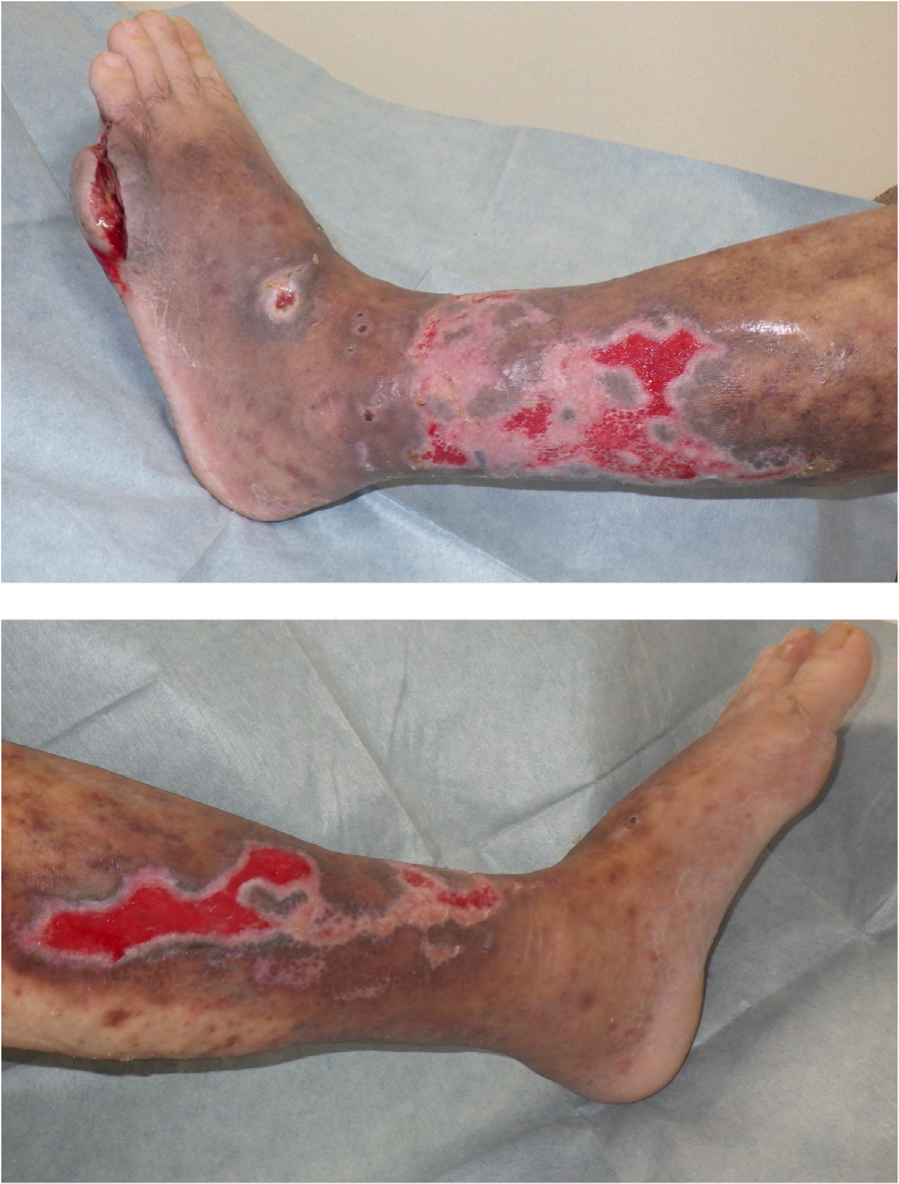
Beginning of EPIFIX® treatment. Due to the refractory nature of the congestive ulcer, treatment with EPIFIX® was initiated 56 days after ulcer onset and reapplied weekly to the ulcer.

**Figure 3:**
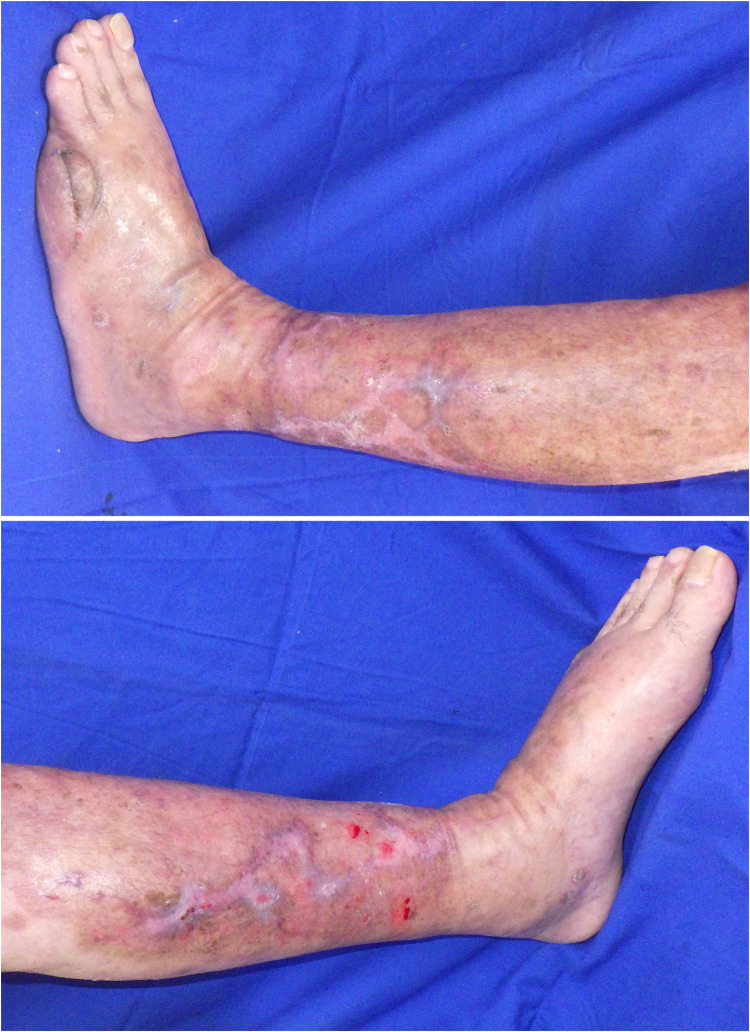
Conclusion of EPIFIX® treatment. After four applications, the area of the ulcer was reduced by 92 % within 3 months of its onset.

An 80-year-old male patient with a history of exertional angina pectoris, and type 2 diabetes mellitus had been under dermatologic care for systemic psoriasis vulgaris and venous stasis dermatitis in both lower limbs since the age of 73. His psoriasis was well-managed with topical medications and an oral phosphodiesterase type 4 inhibitor. On the 35th day before hospitalization, the patient presented to the emergency department with fever and mobility impairment due to pain and admitted for further treatment. Physical examination revealed no other sources of fever besides the lower extremities. The patient was diagnosed with cellulitis and congestive ulceration, and cefazolin treatment was initiated. Daily wound cleansing, topical application of sulfadiazine silver (Geven®, produced by Tanabe Mitsubishi Pharma Corporation, Japan), and the use of an elastic bandage led to a reduction in necrotic tissue; however, the ulcer size remained unchanged, prompting transfer to our department on the 42nd day for specialized management. The left lower leg exhibited an ulcer, approximately 12 × 16 cm in size, characterized by yellowish-brown necrotic tissue with erythema around the ulcer (Figure 1). Laboratory findings showed a white blood cell count of 8400/μL (88.7 % neutrophils), a C-reactive protein (CRP) level of 3.3 mg/dL, and no other abnormal values. The wound cultures were negative. No reduction in transcutaneous oxygen pressure was observed around the ulcer, and the ischaemic limb test results were negative. After admission to our department, daily wound cleansing, topical sulfadiazine silver application, and surgical debridement led to nearly complete removal of the necrotic tissue. By day 56, the CRP level decreased to 0.7 mg/dL, indicating cellulitis improvement. However, the ulcer persisted at approximately 5–10 cm (Figure 2). Consequently, treatment with dHACM (EPIFIX®) was initiated. The ulcer size decreased significantly, and epithelialization progressed rapidly. Skin grafting was considered unnecessary, and conservative treatment was continued. On the 93rd day, the patient was transferred to another facility, where he remained well with no recurrence of ulceration (Figure 3).

## Discussion

The prevalence of psoriasis in Japan is estimated to be 0.1–0.3 % of the population, with over 90 % of patients presenting with plaque psoriasis. Other forms include guttate, erythrodermic, and generalized pustular psoriasis. Psoriasis vulgaris typically presents as well-demarcated erythematous plaques with thick scales. Histopathologically, psoriasis is characterized by epidermal hyperplasia and accelerated mitotic activity in epidermal cells. The underlying pathology involves overexpression of Th17 cells that produce IL-17, leading to increased epidermal cell proliferation. Primary treatment modalities include topical therapy with fixed-dose corticosteroids and vitamin D, narrowband UVB phototherapy, retinoids (vitamin A derivatives), immunosuppressive agents, PDE4 inhibitor, and biologics targeting TNFα, IL-23, and IL-17.

EPIFIX®, introduced in Japan in 2023, is a wound dressing derived from processed and dehydrated amniotic chorioallantoic membrane components sourced from human placenta. It contains an extracellular matrix with over 300 cytokines and growth factors. Clinically utilized in Europe and the United States, both in vitro and in vivo studies have documented its wound healing effects. In Japan, it has also been approved for diabetic foot and venous stasis ulcers that are unresponsive to conventional therapy for at least 4 wk. The conventional TIME framework, which stands for: T, “Tissue non-viable and deficient”; I, “Infection and inflammation”; M, “Moisture imbalance”; and E, “Edge of wound non-advancing or undermined.”), has been expanded to include “R” (Repair and Regeneration) and “S” (Social and patient-related factors). Unlike the original TIME concept, the TIMERS framework expands upon the principles of wound healing by incorporating regeneration and social/patient-centered considerations, emphasizing the significance of individualized care and long-term healing strategies. Among these, dHACM is regarded as highly effective for addressing the “R” component, with promising efficacy for chronic, refractory ulcers.

Comorbidities in cases where dHACM has been proven effective include pyoderma gangrenosum,^2^ infantile haemangioma,^3^ and post-Mohs surgery defects.^4^ While one report exists on using gamma-irradiated amniotic membranes for psoriasis vulgaris,^5^ this is the first report of using dHACM for such conditions. Key regulators of wound healing contained in dHACM include TGF-β, FGF, and PDGF.^6^^,^^7^ dHACM has demonstrated efficacy against immunosuppressive factors such as IL-6, IL-8, MCP-1, MIP-1β, and RANTES, thereby enhancing adipose-derived stem cell activity and promoting wound healing.^8^ Notably, 62 % of patients with venous leg ulcers achieved over 40 % epithelialization after 4 wk of dHACM use.^9^ In this case, dHACM application reduced the ulcer area significantly from 50.0 cm² to 4.0 cm² within 4 wk. Initially, we considered split-thickness skin grafting; however, after dHACM application, wound contraction was substantial and satisfactory epithelialization was achieved through conservative management alone. Unlike skin grafting, dHACM offers the major advantage of facilitating healing in a minimally invasive manner, thereby avoiding donor-site morbidity.

Psoriasis vulgaris is a chronic and refractory skin disorder. Secondary infections can complicate ulcer management because increased skin fragility and persistent inflammation increase the risk of bacterial infection. Given the extensive array of treatment options currently available, a careful multimodal treatment approach should be tailored for each case based on clinical presentation, comorbidities, individual patient characteristics, and treatment priorities. As seen in the present case, for venous stasis ulcers associated with cellulitis, early-stage treatment should incorporate dHACM guided by the TIMERS framework, with a focus on adequate reduction of inflammation.

This case suggests that dHACM may promote wound healing in patients with refractory venous stasis ulcers complicated by psoriasis vulgaris. The use of dHACM represents a significant treatment option in such cases, and its potential as a regenerative therapy is expected to be both highly effective and improve outcomes compared to conventional treatments.

## Funding

None.

## Ethical approval

Not required.

## Conflict of interest

None.
